# SAST-GCN: Segmentation Adaptive Spatial Temporal-Graph Convolutional Network for P3-Based Video Target Detection

**DOI:** 10.3389/fnins.2022.913027

**Published:** 2022-06-02

**Authors:** Runnan Lu, Ying Zeng, Rongkai Zhang, Bin Yan, Li Tong

**Affiliations:** ^1^Henan Key Laboratory of Imaging and Intelligent Processing, People’s Liberation Army of China (PLA) Strategic Support Force Information Engineering University, Zhengzhou, China; ^2^Key Laboratory for Neuroinformation of Ministry of Education, School of Life Sciences and Technology, University of Electronic Science and Technology of China, Chengdu, China

**Keywords:** brain-computer interface (BCI), electroencephalography (EEG), P3 detection, graph convolutional neural networks (GCN), style-based recalibration module (SRM)

## Abstract

Detecting video-induced P3 is crucial to building the video target detection system based on the brain-computer interface. However, studies have shown that the brain response patterns corresponding to video-induced P3 are dynamic and determined by the interaction of multiple brain regions. This paper proposes a segmentation adaptive spatial-temporal graph convolutional network (SAST-GCN) for P3-based video target detection. To make full use of the dynamic characteristics of the P3 signal data, the data is segmented according to the processing stages of the video-induced P3, and the brain network connections are constructed correspondingly. Then, the spatial-temporal feature of EEG data is extracted by adaptive spatial-temporal graph convolution to discriminate the target and non-target in the video. Especially, a style-based recalibration module is added to select feature maps with higher contributions and increase the feature extraction ability of the network. The experimental results demonstrate the superiority of our proposed model over the baseline methods. Also, the ablation experiments indicate that the segmentation of data to construct the brain connection can effectively improve the recognition performance by reflecting the dynamic connection relationship between EEG channels more accurately.

## Introduction

Event-Related Potentials (ERP) is a special type of brain-evoked potential. It can reflect the neurophysiological changes in the human brain according to cognitive behavior, thus revealing the processing of sensitive information in the brain (Kutas et al., 1977; Brydges and Barceló, 2018). However, the signal-to-noise ratio of ERP signals is low, and it is difficult to detect these potentials, especially in a single trial. In dynamic video target detection tasks, the dynamic change of the scene has a complex impact on the brain response, which further improves the difficulty of single-trial P3 detection. The single-trial video-induced P3 detection model should be considered from many aspects, which is of great significance to shortening the preparation time of test users and improving the practicability and generalization of the brain-computer interface (BCI) system (Song X. et al., 2020).

The previous work used the traditional machine learning technology and the classifier based on a neural network for single-trial EEG classification. The classical machine learning algorithms such as K-Nearest Neighbor (KNN) (Schlögl et al., 2005), Naive Bayes (NB) (Speier et al., 2012), Support Vector Machine (SVM) (Thulasidas et al., 2006), Discriminative Canonical Pattern Matching (DCPM) (Xiao et al., 2020) depend largely on feature engineering and feature selection, which requires a lot of expert knowledge. In recent years, deep learning methods have been widely used in EEG signal classification due to their strong representation learning ability (Anumanchipalli et al., 2019). Various deep learning methods have been used for EEG recognition, and some of them have achieved good results (Lawhern et al., 2016; Lan et al., 2021; Ma et al., 2021). To achieve better generalization performance of deep neural networks, Lawhern et al. (2016). proposed a compact convolutional neural network EEGNet, which extracts the frequency-domain, space-domain, and time-domain features of EEG signals through three different convolutional kernels and achieves a good detection effect on P300 signals. Meanwhile, some scholars studied the application of recurrent neural network (RNN) to ERP classification to capture the time series information hidden in the ERP signals. An Indian scholar proposed convolutional long short term memory (ConvLSTM) (Joshi et al., 2018), which combines a convolutional neural network and a long-short-term neural network. ConvLSTM extracts the spatial characteristics of EEG signals by the CNN network and learns the temporal variation of EEG signals through LSTM to obtain a better ERP classification effect than the single CNN network.

However, EEG data has spatial and temporal characteristics. The spatial structure is related to the different EEG sensors placed on the scalp of the subjects, while the temporal structure is implicit because it obtains voltage values at each instant (Joshi et al., 2018). Thus, P300 has similar temporal characteristics but different spatial characteristics, and the spatial connection should not be ignored in EEG data. However, the recent commonly used deep learning methods require grid data as input (itself or converted into a similar image representation) and ignore the connection between the spatial regions of the brain (Wang et al., 2021). Since the brain region is in a non-Euclid space, the graph is the most suitable data structure to describe brain connections.

The graph neural network (GNN) is a neural network that can be directly applied to the graph data structure. The graph convolutional neural network (GCN) is a type of GNN, which uses convolution operations. It captures the dependency relationship in the graph according to the information transmission between the nodes in the graph, and then it performs end-to-end calculations on the graph data (Cai et al., 2017; Zhou et al., 2018). The emerging GCN has shown potential in modeling multi-channel signals through graphs in a non-Euclid space. Instant applications include image classification (Bruna et al., 2013; Monti et al., 2017; Chen et al., 2019), node classification (Kipf and Welling, 2016), action recognition (Yan et al., 2018; Zhang et al., 2019) and traffic flow forecasting (Yu et al., 2018; Diao et al., 2019).

By relating each EEG channel to a node of the graph and the connections between the channels to the edges of the graph, some scholars proposed to use GCN for EEG data classification (Wagh and Varatharajah, 2020). Song T. et al. (2020) first used Dynamical Graph Convolutional Neural Network (DGCNN) for EEG emotion recognition. The network can construct graph data more in line with the state of the brain activity by learning the relationship between different channels, and it achieves good performance. Zhong et al. (2020) proposed a regularized convolutional network (RGNN), which considers the global and local relationships of different EEG channels. The topological structure of the EEG data is considered by the above studies, but the ERP signal has obvious time-domain characteristics. Therefore, Yan et al. (2018) introduced the spatial-temporal graph convolutional network (GCN). Sun et al. (2021) proposed an adaptive spatial-temporal GCN for classifying motor imagery EEG data. Meanwhile, they extracted the spatial-temporal features synchronously through one-dimensional convolution in the time domain and graph convolution in space. Jia et al. (2020) proposed GraphSleepNet for automatic sleep stage classification. The model uses a graph convolution to extract spatial features and a time convolution to capture the conversion rules between sleep stages. Also, it adaptively learns the intrinsic connections between different EEG channels to best serve the classification of sleep stages.

At present, the graph connection representation in EEG data based on graph convolution only uses a static connection mode, which is not consistent with the fact that the brain network connection changes from time to time. Especially, for video-induced P3, the neural response can be divided into three stages, i.e., information integration, decision process, and neuronal response (Desmedt, 1980; Song et al., 2021b). Therefore, this paper proposes a segmentation adaptive spatial-temporal GCN (SAST-GCN) for single-trial video-induced P3 detection based on the spatial-temporal structure of multi-channel EEG signals. According to the neural processing mechanism for video targets, the EEG data are divided into stages, and the corresponding graph representations are constructed. Based on this, the spatial-temporal characteristics are extracted by spatial-temporal graph convolution.

The main contributions of this paper are as follows:

(1) A segmented constructed graph method is proposed. Compared with the unified static graph, the segmented constructed graph can improve network performance by 4.42% (F1-score).

(2) SAST-GCN can adaptively determine the importance of adjacent nodes and extract spatial-temporal domain features synchronously in a unified spatial-temporal GCN layer.

(3) The style-based recalibration module (SRM) is introduced to realize adaptive calibration of the intermediate feature map, which emphasizes the information related to P3 components and ignores other information.

## Dataset

In this section, the experimental paradigm and data acquisition and preprocessing methods are introduced.

### Experiment Paradigm

The data from Song X. et al. (2020) EEG-based video target detection experiment are used in this study. In their experiment, 34 healthy college students were recruited, with an average age of 25. All subjects signed informed consent before the experiment. The experiment was approved by the Ethics Committee of Henan Provincial People Hospital.

**FIGURE 1:**
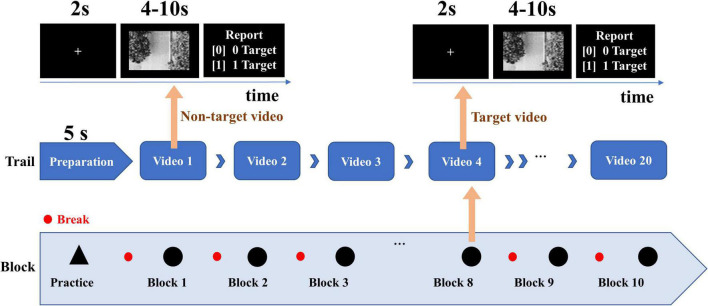
Experimental paradigm of UAV video target detection (Song et al., 2021a).

The video stimulus comes from a UAV aerial video. The experimental paradigm of video target detection based on EEG signals is shown in Figure 1. The drone flew along a wide street and was videotaped. The experiment included 200 video clips, of which 100 video clips containing vehicles were deviation videos, and 100 video clips without vehicles were standard videos. The 200 video clips were randomly arranged into 10 blocks, and each block includes 20 video clips with a duration of 4–10 s. In the experiment, the participants were asked to quickly find the vehicles in the video and ignore other interference information. At the end of each video, feedback on whether the vehicle appeared in the video or not was given *via* keystrokes.

### Data Acquisition and Preprocess

The g. HIamp EEG acquisition system provided by the Austrian g.tec Company was used to collect EEG signals, which contained 61 channels of effective EEG signals. The online sampling frequency of the EEG system was 600 Hz, the band-pass filter was 0.01˜100 Hz, and the notch frequency was 50 Hz. In the experiment, a 0.1–30 Hz band-pass filter was used to further filter the signal, the independent component analysis method was adopted to remove the EOG signal, and the signal was downsampled to 100 Hz. Meanwhile, the error key video corresponding to the EEG signal was removed. To provide reliable sample labels, the bias samples were intercepted from the EEG signals induced by the biased video, and the standard samples were intercepted from the EEG signals induced by the standard video. The interception method of the target sample is: the targets appearing for 1,500 ms are intercepted. Since each deviation video contains only one vehicle, a deviation video can only provide a reliable deviation sample. The standard video capture method is: the standard samples with an overlap length of 1,500 ms are captured from the whole EEG evoked by the standard video. In this way, each standard video can provide multiple standard samples. Using the ERP alignment method proposed in the previous study (Song X. et al., 2020), these samples were aligned with ERP templates to extract 1,000-ms aligned single-trial EEG signals, and the signals with an amplitude greater than 100 μV were discarded. After the above preprocessing, 300–500 valid single-trial signals can be obtained for each participant, and the size of the single-trial signal matrix is 61 × 100. The ratio of deviation sample to standard sample ranges from 1:4 to 1:4.5.

## Methods

In this section, the model architecture, implementation details of the SAST-GCN model, and the training and testing methods are introduced.

### Model Architecture

**FIGURE 2:**
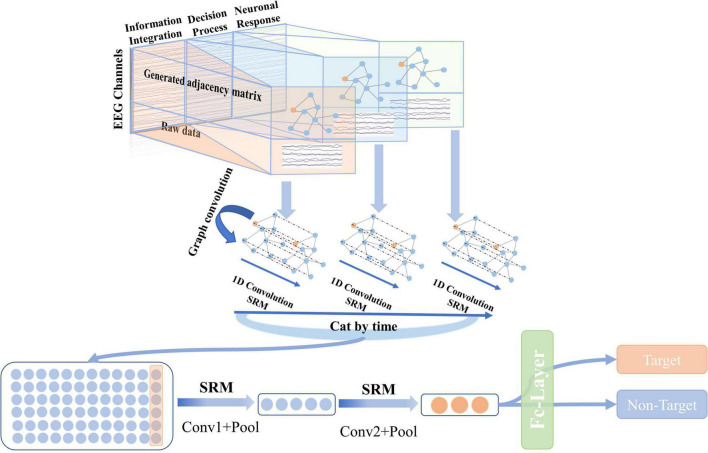
The overall architecture of the proposed SAST-GCN. The model consists of three parts: the segmented adjacent matrix construction module, the spatial-temporal graph convolution module, and the classification module.

The overall architecture of the proposed SAST-GCN is shown in Figure 2. The SAST-GCN model consists of three blocks that work in order: (1) The original data is represented by graphs in time dimension according to the neural mechanism of brain processing videos, and the initial adjacency matrix is obtained by Pearson coefficient. (2) Spatial graph convolution is combined with temporal convolution to extract spatial and temporal features. A style-based recalibration module is added to enhance the representation ability of CNN. (3) Dimension reduction is performed by two-layer standard convolution and pooling operations, and classification is performed by the full connection layer.

#### Segmented Graph Construction

##### Data Segmentation

Studies have shown that the human brain has obvious temporal characteristics for video target perception. Video target-induced P3 can be divided into three stages: (1) information integration (about 0–200 ms), (2) decision process (about 200 ms to P300 latency), and (3) neuronal response (after P300 peak latency). The brain response intensity gradually increases and then weakens; correspondingly, the brain network connection gradually enriches and then weakens. Therefore, it is necessary to construct an adjacency matrix according to the continuous change of the brain connection network (Desmedt, 1980; Wang et al., 2014; Song et al., 2021b).

**FIGURE 3:**
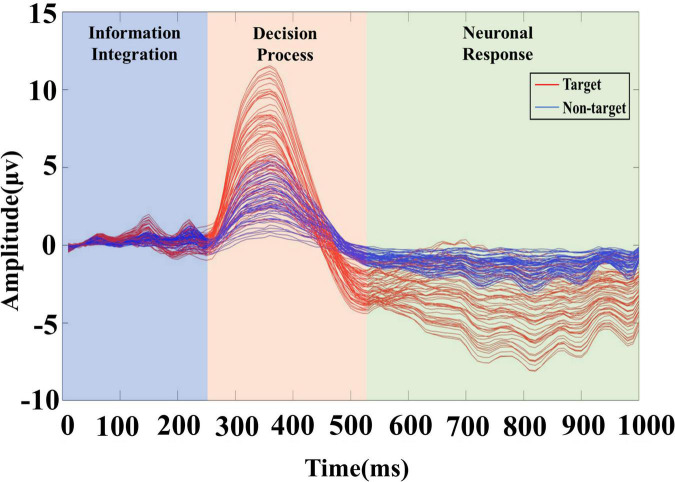
The brain response of the video target. After overlaying and averaging the EEG signals of all trials according to the channel, the red curve represents the target trial data, and the blue curve represents the non-target trial data.

To further determine the division range through the changes in the brain neural response, this paper draws the average ERP response of each channel to the neural response of the task target within 1 s in the video, as shown in Figure 3. Each curve in the figure represents the average ERP signal of a channel, showing the target and non-target signals of 61 channels. According to the superimposed ERP waveform, the EEG response induced by the task target has obvious time characteristics, and the signal can be divided into three stages. Therefore, this paper further clarifies the specific time scale of each stage. Positive P1 and P2 components appear at about 130 and 230 ms, so the range of 0–250 ms is adopted to represent the information integration stage. The attention and information integration of task objectives are performed by the brain. There is an obvious P3 peak around 350 ms, so the range of 250–520 ms is adopted to represent the decision processing stage of the task target. Besides, the range of 520–1,000 ms is adopted to represent the neuronal response stage.

##### Graph Construction

Inspired by the application of graph convolution neural networks in image processing (Rivet et al., 2015; Levie et al., 2017; Fu et al., 2020), this paper transforms the video-induced P3 detection problem into the graph data processing problem. In the proposed graph representation, each EEG channel is represented as a node, and the functional relationship between the two channels is represented as an edge of the graph (Song T. et al., 2020). The value of the edge indicates the closeness of the functional relationship. The larger the edge value, the closer the functional relationship between the two channels.

To represent the connection between electrodes, this paper introduces the Pearson coefficient to calculate the connection between electrodes. The calculation formula is as follows Eq. (1) (Bao et al., 2022):


(1) $$S\left(i,j\right)=\left|PCC\left(x_{i},x_{j}\right)\right|=\left|\frac{cov\left(x_{i},x_{j}\right)}{\sigma_{x_{i}}\sigma_{x_{j}}}\right|$$

where *i, j* = 1, 2, ……, *n*, and *n* is the number of channels for EEG signals; *x*_*i/j*_ is the *i*/*j*-th channel EEG signal; *cov*() means covariance.

To ensure the sparsity of the matrix, a threshold α is defined in this paper. When *S*(*i*, *j*) ≥α, the *i*-th node is connected to the *j*-th node in the constructed graph and vice versa; when *S*(*i*, *j*)<α, it was considered unconnected. Thus, the structure of a graph can be represented as:


(2) $$A_{ij}={\{}_{0,s\left(i,j\right)<\alpha }^{1,s\left(i,j\right)\geq \alpha }$$

where, *A* represents the connection between electrode channels.

**FIGURE 4:**
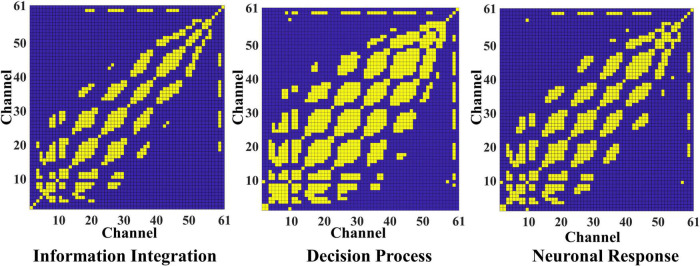
The adjacent matrix of the information integration, decision process, and neuron response. The yellow block represents a connection between the corresponding electrodes, and the blue block represents no connection.

According to the time division of the segmentation and the graph construction formula, the initial adjacency matrix of the brain connection in three stages is calculated, respectively, and the three matrixes are obtained to represent the brain network connection of video target-induced P3. Need to say, here we set the threshold α to 0.8, for the adjacency matrix of each stage, we retain 15.83, 28.57, and 19.59% of the node connection, so the average sparsity is about 20.00%. This is also a widely accepted degree of sparsity in computing brain network connection. In Figure 4, the yellow block represents connections between nodes, and the blue block rep resents no connections. All connections are bidirectional, so all matrices are symmetric. It can be seen that the connections between the electrodes in different stages are distinguished, and the brain connections are the most abundant in the decision process stage. This is because the decision process stage is to identify the integrated information, and the specific brain area communication of different task-related networks is crucial to the generation of P3. Therefore, the activation intensity of the brain and the richness of brain network connections are the highest in this stage.

#### Adaptive Spatial-Temporal Graph Convolution With Style-Based Recalibration

Spatial-temporal graph convolution combines space graph convolution and time standard convolution, which is used in this paper to extract space and time features simultaneously. Spatial features are extracted by collecting information from neighbor nodes of each electrode node, and temporal features are extracted by time dependence in the time dimension.

##### Spatial Graph Convolution

GCN can be implemented in two approaches, i.e., spectral approaches and spatial approaches. However, spectral GCN has great limitations, such as failing to deal with directed graphs and large graphs, leading to poor scalability. Currently, spatial approaches have developed rapidly in recent years because of their good efficiency, flexibility, and versatility. Therefore, this paper adopts spatial graph convolution.

As for graph construction, in the case of a single frame, the adjacency matrix Λ is used to represent the internal connection between nodes, and the weight matrix *W* is formed by stacking the weight vectors of multiple output channels. To avoid the product multiplication in the adjacency matrix and feature matrix to change the original distribution of features, this paper conducts a normalization processing on A. Let A be multiplied with the degree matrix labeled –1 and further split into a symmetric matrix and a normalized matrix labeled −½. Therefore, the propagation of GCN is realized by the following formula Eq. (3) and Eq. (4) (Kipf and Welling, 2016):


(3) $$G^{\left(k\right)}=D^{-\frac{1}{2}}\text{Λ}D^{-\frac{1}{2}}G^{\left(k-1\right)}W^{k-1}$$

(4) $$G^{\left(0\right)}=X$$

where, Λ(Λ ∈ ℝ^*N*×*N*^) denotes the adjacency matrix of the data; *D* is the degree matrix of Λ; *G*^(*k*)^ and *G*^(*k*−1)^ represent the output of the next layer and the input of the upper layer, respectively; *k* ∈ 1, 2…*N*, and *N* is the total number of layers of the network; *W* is the weight matrix, and *X* is the input data.

In the actual implementation, the input feature map can be expressed as a tensor with a dimension of (*C, V, T*). Graph convolution is realized by multiplying a 1×Γ two-dimensional convolution result with a normalized adjacency matrix $D^{-\frac{1}{2}}\Lambda D^{-\frac{1}{2}}$ on the second dimension.

In the spatial-temporal graph convolution, the normalized adjacency matrix $D^{-\frac{1}{2}}\Lambda D^{-\frac{1}{2}}$ (Λ ∈ ℝ^*N*×*N*^) is used to represent the connection relationship between nodes. For an undirected graph, if there is a connection between nodes, Λ_*ij*_ is equal to 1; otherwise, Λ_*ij*_ is equal to 0. However, for EEG data, neighbor nodes have different influences on central nodes, and the weights of edges are unknown, so it is not suitable to describe the EEG data by undirected graphs. Therefore, this paper proposes an adaptive graph convolution to adaptively learn edge weights. To ensure the adaptability of the graph representation, a learning weight matrix *M*is introduced, and it is multiplied with the normalized adjacency matrix, i.e., $M⊙D^{-\frac{1}{2}}\Lambda D^{-\frac{1}{2}}$, where ⊙ means element-wise product between two matrices, and *M* is initialized to consist of all 1 s. In this way, the calculation of adaptive graph convolution can be defined as Eq. (5) (Yan et al., 2018):


(5) $$G^{\left(k\right)}=D^{-\frac{1}{2}}\Lambda D^{-\frac{1}{2}}⊙MG^{\left(k-1\right)}W^{k-1}$$

##### Time Convolution

For time-varying graph data, it is necessary to extract features in the time domain, so this paper adds a one-dimensional standard convolution to extract time-domain features. Specifically, a standard 1D convolution layer is used to extract time-domain features in the current time domain and send them to the graph convolution operation to fully extract spatial features. Then, the time-domain convolution can be defined as:


(6) $$H^{\left(k\right)}=ELU{\text{Φ}}^{*}{\hat{H}}^{\left(k-1\right)}$$

where, *ELU* is the activation function, Φ represents the convolution kernel parameter, and * is the standard convolution.

Thus, the whole spatial-temporal graph convolution layer can be represented as:


(7) $$\chi^{l}=ELU\left(\left(\Phi_{g}\overset{⌢}{{}^{*}}\left(ELU\left(\Phi^{*}\chi^{l-1}\right)\right)\right)\right)$$

where, Φ_*g*_ represents the convolution kernel of the graph convolution.

##### Style-Based Recalibration

Based on the spatial-temporal graph convolution, this paper adds a style-based recalibration module and a graph adaptive mechanism to improve the spatial-temporal feature extraction ability of spatial-temporal graph convolution for video-induced P3.

**FIGURE 5:**
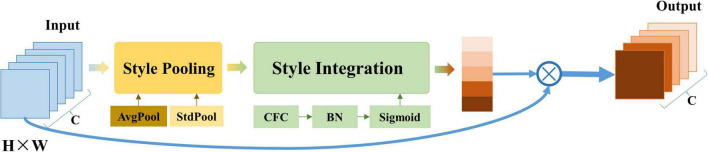
The architecture of the style-based recalibration module. This module is mainly composed of two parts: style pooling and style integration. AvgPool refers to global average pooling; StdPool refers to global standard deviation pooling; CFC refers to the channel fully connected layer; BN refers to batch standardization.

The style-based recalibration module is a simple and effective architecture unit. This module was first proposed by Lee et al. (2019), and it can adaptively recalibrate intermediate feature maps by using their style information. As shown in Figure 5, the module consists of two main components: style pooling and style integration. Style pooling extracts style features from each channel by summarizing the feature responses across spatial dimensions (including the parallel processing of global average pooling and global standard deviation pooling) and splicing the pooling data. Subsequently, style integration uses style features to generate specific style weights through channel-based operations, including forming a set of weight vectors through CFC (Channel Full Connection layer), BN (Batch Normalization), and sigmoid activation functions. Then, the input data is multiplied with the weight in the feature dimension to emphasize or hide information.

It can be seen from the operation of SRM that it can recalibrate the intermediate feature map adaptively by using its style information. SRM first extracts the style information from each channel of the feature map through style pooling and then estimates the recalibration weight of each channel through the style integration unrelated to the channel. SRM can effectively improve the representation ability of CNN by incorporating the relative importance of each style into the feature map with fewer parameters, which performs better than the traditional attention mechanism SENet.

After adding a layer of SRM operation before one-dimensional time-domain convolution, the whole spatial-temporal graph convolution layer can be represented as:


(8) $$\chi^{l}=ELU\left(\left(\Phi_{g}\overset{⌢}{{}^{*}}SRM\left(ELU\left(\Phi^{*}\chi^{l-1}\right)\right)\right)\right)$$

where, *SRM*(⋅) representing the style-based recalibration operation.

#### Classification Module

In the classification module, the extracted features of the segmented spatial-temporal convolution are spliced in the time domain, and the classification is made according to the output of the two convolution layers and one fully connected layer. The first convolution layer includes a convolution operation with a convolution kernel size of (1, *N*) and an average pooling operation with a pooling size of (*K*_1_, 1). *N* is the number of electrodes, so the output of the first convolution layer is (*F*_1_, *T*/*K*_1_, 1). The second convolution layer includes a convolution operation with a convolution kernel size of (1, 1) and an average pooling operation with a pooling size of (*K*_2_, 1) to extract and integrate features and reduce the dimension. To further extract important features, the SRM module is added to the convolution operation here. Each convolution layer can be represented as:


(9) $$C^{k}=Pool\left(ELU\left(SRM\left(\Phi^{*}C^{\left(K-1\right)}\right)\right)\right)$$

where, *Pool*(⋅) refers to the pooling operation, *C*^0^ = χ*^l^*.

Finally, the prediction results are output by a full connection layer. Therefore, the classification of input EEG signals is:


(10) $$\hat{y}=softmax\left(FC\left(C\right)\right)$$

where, C refers to the output after the convolution layer; *FC*(⋅)refers to the full connection layer operation; $\hat{y}\in {\mathbb{R}}^{2}$ is a two-class prediction label.

Cross-entropy is used here to measure the difference between the real probability distribution and the predicted probability distribution to optimize the model.


(11) $$ℒ=cross\_entropy\left(y,\hat{y}\right)$$

where, *y* refers to the real label of the sample, and *cross*_*entropy*(⋅) refers to the cross-entropy calculation.

### Implementation Details of the Segmentation Adaptive Spatial-Temporal Graph Convolutional Network Model

The specific algorithm of SAST-GCN is shown in Algorithm 1. The raw data is divided into three segments, and the adjacency matrix is constructed. Then, the features are extracted through the spatial-temporal graph convolution layer with the SRM. After splicing, the dimension is reduced through the standard convolution layer and average pooling. Finally, the prediction label is output by the full connection layer.

**ALGORITHM 1:** The training process of SAST-GCN.

**Input:** A labeled training data set $\left\{X,Y\right\}={\left\{x_{i},y_{i}\right\}}_{i=1}^{N}$, the maximum number of training epochs T; the initialize adjacency matrix *A*_1_, *A*_2_, *A*_3_; the learnable weight matrix *M*_1_, *M*_2_, *M*_3_;
**Output:** The learned adjacency matrix ${\hat{A}}_{1},{\hat{A}}_{2},{\hat{A}}_{3}$, the model parameter Θ for SAST-GCN and the predicted label $\hat{y}$.
**Step1:** Initialize the model parameters Θ in SAST-GCN model. Set iteration unit iter = 1;
**Step2:** while *iter* < *T***do**
**Step3:** Splitting single trial data into three segments in time domain, *X*_1_, *X*_2_, *X*_3_;
**Step3:** for *k* = 1, …, /**do**
**Step4:** Calculate the *k*-th spatial-temporal graph convolution *via* Eq. 8;
**Step5:** Concatenate the features χ_1_, χ_2_, χ_3_ after spatial-temporal graph convolution in time domain;
**Step5:** for *k* = 1, …, /**do**
**Step6:** Calculate the *k*-th convolution layer *C^k^via* Eq. 9;
**Step7:** Calculate the prediction label $\hat{y}$ *via* Eq. 10;
**Step8:** Update the learnable weight matrix *M*_1_, *M*_2_, *M*_3_ and the model parametersΘ*via* optimizer according to the cross entropy loss Eq. 11;
**Step9:** *iter* = *iter* + 1;
**Step10:** end while

Considering that the amount of target detection EEG data is too small, this paper aims to design a compact architecture for SAST-GCN so that: (1) it can alleviate overfitting problems and (2) can achieve better efficiency. Meanwhile, the graph convolution network should be superimposed within 5 layers; otherwise, the performance will be affected. After a small number of trial-and-error experiments, it was observed that SAST-GCN achieved high accuracy under a two-layer graph convolution layer and two-layer convolution layer plus a one-layer full connection layer. The detailed description of the SAST-GCN model is shown in Table 1.

**TABLE 1:** SAST-GCN architecture.

Block	Layer	Kernel size	Stride	Input	Output	Activation
ST-graph convolution	Input				(1,T,C)	
	Segmentation			(1,T,C)	(1,T1,C) (1,T2,C) (1,T3,C)	
	STGCN1-TCN	(63,1)	1	(1,T1,C) (1,T2,C) (1,T3,C)	(8,T1,C) (8,T2,C) (8,T3,C)	ELU
	STGCN1-GCN	(1,1)	1	(8,T1,C) (8,T2,C) (8,T3,C)	(8,T1,C) (8,T2,C) (8,T3,C)	ELU
	STGCN2-TCN	(63,1)	1	(8,T1,C) (8,T2,C) (8,T3,C)	(16,T1,C) (16,T2,C) (16,T3,C)	ELU
	STGCN2-GCN	(1,1)	1	(16,T1,C) (16,T2,C) (16,T3,C)	(16,T1,C) (16,T2,C) (16,T3,C)	ELU
	Concatenation			(16,T1,C) (16,T2,C) (16,T3,C)	(16,T,C)	
Standard convolution	Conv1	(1,61)	1	(16,T,C)	(32,T,1)	ELU
	Avg_pool1	(5,1)	1	(32,T,1)	(32,T/5,1)	
	Conv2	(1,1)	1	(32,T/5,1)	(64,T/5,1)	ELU
	Avg_pool2	(5,1)	1	(64,T/5,1)	(64,T/25,1)	
Classifier	Reshape			(64,T/25,1)	(64×T/25×1)	
	Full-connection			(64×T/25×1)	2	Softmax

*Where, T refers to the number of time points in all stages; C refers to the number of channels; T1, T2, and T3 refers to the number of time points in the formation integration, decision process, and neural response stages, respectively.*

### Training and Testing Method

The SAST-GCN network is implemented based on the PyTorch framework. The input signal size is 61×100 (number of channels × number of sampling points), and the sampling frequency is 100 Hz. The data segmentation in SAST-GCN is 61 × 25 (0–250 ms), 61 × 27 (250–520 ms), and 61 × 48 (520–1,000 ms), respectively. All dropout rates are 0.5. In the experiment, the Adam optimizer was used to fit the model. The learning rate was set to 0.0003, 100 rounds of iterative training were performed, and the batch size was *K* = 128.

The leave-one method is adopted to verify the proposed method. Each training set includes the data of 33 subjects, and the test set includes the EEG data of the remaining one subject. To ensure the sample balance during training, the non-target data in the training set was downsampled to keep balance with the target data.

## Results and Analysis

This section introduces the evaluation of the effectiveness and advancement of the proposed model on the data sets described in section “Dataset.”

### Overall Performance

**FIGURE 6:**
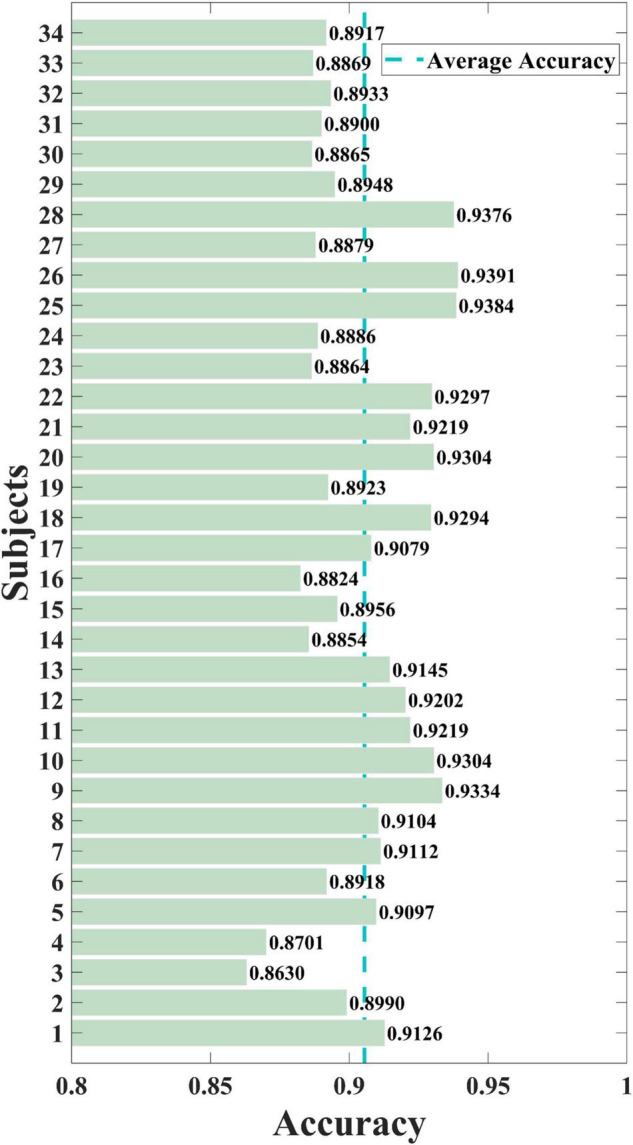
The overall performance of the SAST-GCN model. Average Accuracy refers to average accuracy of all subjects, which is 0.9055.

Each participant was tested, and the cross-subject results of the training set were obtained. As shown in Figure 6, the proposed SAST-GCN model performs well on the whole dataset, with the lowest accuracy of 86.30% in Sub3 and the highest accuracy of 93.91% in Sub26. The average accuracy of the model is 90.55%, and the standard deviation is 2.08%. The accuracy of 17 subjects exceeds the average accuracy. These results show that the proposed SAST-GCN model is effective and stable for video-induced P3 detection.

### Method Comparison

To further explore the benefit of the spatial-temporal structure of SAST-GCN, eight widely used competitive EEG models were taken for performance comparison, including KNN, Random Forest, Support Vector Machine, Naive Bayes, AdaBoost, Fusion of Traditional Algorithms, EEGNet, and CNN-LSTM. Meanwhile, the fusion of traditional algorithms was considered, i.e., fusing KNN, RF, SVM, Naive Bayesian, and AdaBoost by a voting method. EEGNet is an EEG signal classification network proposed by Lawhern, and it has high recognition accuracy and good generalization performance. CNN-LSTM represents the combination of CNN and LSTM by the method proposed in the previous study (Abibullaev and Zollanvari, 2021), which extracts EEG features by using the representation ability of the convolution layer and the ability to capture temporal dependencies of LSTM.

The average accuracy, F1-score, precision, recall, Floating Point Operations (FLOPs) and parameters of each method are presented in Table 2. The results show that compared with other baseline methods, our SAST-GCN achieves the best performance in three evaluation indicators.

**TABLE 2:** The accuracy, F1-score, precision, recall and complexity of different methods.

Model	Accuracy	F1-score	Precision	Recall	FLOPs	Parameters
KNN	0.6776	0.3728	0.2897	0.5323		
RF	0.7143	0.4236	0.3381	0.5834		
SVM	0.7856	0.5351	0.4554	0.6864		
Naive Bayes	0.6863	0.4017	0.3114	0.6072		
AdaBoost	0.6762	0.3915	0.3147	0.5723		
Fusion of traditional algorithms	0.7541	0.4731	0.3983	0.6251		
EEGNET	0.8274	0.6195	0.5536	0.7390	0.837G	1.2K
CNN-LSTM	0.8509	0.5647	0.6145	0.5418	61.34G	282.2K
SAST-GCN	**0.9055**	**0.7042**	**0.7003**	**0.7100**	**3.72G**	**23.8K**

*The bold values represent the results of the proposed method.*

The traditional machine learning methods (KNN, RF, SVM, Naive Bayes, and AdaBoost) and their fusion algorithms cannot learn complex spatial-temporal features well and cannot obtain satisfactory results. However, the existing deep learning models, such as EEGNet and CNN-LSTM, can extract spatial or temporal features. Therefore, they achieve better performance is better than those based on traditional machine learning.

Although CNN and RNN have high precision, their limitation is that the input of the model must be grid data, and the connection between regions is ignored. Since the brain region is in a non-Euclid space, the spatial relationship of signals cannot be fully and accurately reflected by flattening EEG channels on irregular grids into two-dimensional representations with regular grids. The graph is the most suitable data structure to represent the connection, The proposed SAST-GCN extracts spatial and temporal features based on the segmentation adaptive graph structure to improve performance. Therefore, the SAST-GCN method proposed in this paper is superior to other baseline methods. Its accuracy is 9.44 and 6.42% higher than that of EEGNET and CNN-LSTM, and its F1-score is 13.67 and 24.70% higher than that of EEGNET and CNN-LSTM, respectively.

In order to analyze the possible real-time application and technology transfer scenarios of this method, this paper further analyzes the time complexity and spatial complexity of the model, and compares it with the two baselines (EEGNET and CNN-LSTM). The Floating-Point Operations (FLOPs) is used to measure the time complexity of the model. High time complexity will lead to a large amount of time for model training and prediction, which means the model cannot be quickly verified, improved, and achieve rapid prediction. The parameters are used to measure the space complexity of the model. Due to the limitation of dimension disaster, the more parameters of the model, the more data is needed to train the model, and the EEG data set is usually not too large, which will lead to the problem of overfitting.

It can be seen from Table 2 that FLOPs and Parameters of SAST-GCN are much lower than those of CNN-LSTM, but higher than those of EEGNET, which may be due to the increase of convolution layers. However, considering the improvement of performance, this should be within an acceptable range.

### Ablation Studies

To verify the contribution of each module to the model performance, ablation experiments were conducted on the model, and the results are presented in Table 3.

**TABLE 3:** Ablation studies.

Operation	Accuracy	F1-score	Precision	Recall	FLOPs	Parameters
ASTGCN	0.8787	0.6607	0.6414	0.6812	3.72G	15.3K
ASTGCN+SRM	0.8883	0.6744	0.6816	0.6814	3.72G	15.5K
ASTGCN+SRM + Segmentation	**0.9055**	**0.7042**	**0.7003**	**0.7100**	**3.72G**	**23.8K**

*The bold values represent the results of the proposed method.*

To measure the influence of segmented construction adjacency matrix operation, the segmented construction adjacency matrix operation (ASTGCN + SRM + Segmentation) is further added based on the adaptive spatial-temporal graph convolution (ASTGCN + SRM) with SMR. It can be found that the accuracy, F1-score, precision, and recall are increased by 1.94, 4.42, 2.74, and 4.20%, respectively.

For the whole non-segmented adjacency matrix, the performance of the segmented matrix is excellent. The reason is that the connection of the brain network changes in different processing stages of the brain, so the segmented construction matrix can more accurately reflect the relationship between EEG channels. By contrast, the whole uniform adjacency matrix ignores the dynamic changes in the brain network.

We also conducted an ablation study of complexity. It can be found that after adding SMR, FLOPs are not affected, and the number of parameters increases by only 0.2 K, which also proves that SMR can improve model performance without increasing model complexity. Further, after segmenting, FLOPs are not affected, but the number of parameters increases by 8.3 K due to the addition of spatial-temporal graph convolution layers to the simultaneous convolution of three segments of data.

### Impact of Different Stages

Furthermore, to verify the impact of the information integration, decision process, and neural response stages on target recognition performance, the results of using the data in each of the stages and all stages are compared, as shown in Table 4. It can be observed that the classification accuracy of using the data in the decision processing stage alone is the highest, and the F1-score is 2.45 and 0.81% higher than that of using the data in the formation integration and neural response stages, respectively, which is consistent with the existing studies. These studies show that the cognitive function of the brain reaches the strongest in the decision processing stage. Meanwhile, the connection strength and information conversion ability between the multi-brain regions reach the highest, which is conducive to identifying the integrated information (Li et al., 2018).

**TABLE 4:** Comparison of effects of using the data in different stages on classification results.

Stage of the brain response for classification	Accuracy	F1-score	Precision	Recall	FLOPs	Parameters
Formation integration	0.8772	0.6344	0.6322	0.6521	1.23G	15.1K
Decision processing	0.8836	0.6499	0.6475	0.6594	1.30G	15.1K
Neural response	0.8816	0.6447	0.6378	0.6613	2.00G	15.1K
All stages	**0.9055**	**0.7042**	**0.7003**	**0.7100**	**3.72G**	**23.8K**

*The bold values represent the results of the proposed method.*

However, it is also observed that the classification results of using the data in all stages are significantly better than those of using the data in only one stage. The F1-score of using the data of all stages is 11.00, 8.36, and 9.23% higher than that of using the data in the formation integration stage, decision processing stage, and neural response stage, respectively. There are two reasons for this. One is that when the data in a certain stage are used separately for classification, the amount of data is too small to extract more effective information. Second, the perception of brain processing video targets consists of a series of complex processing processes, involving evidence accumulation across time and space. Therefore, the joint action of all the stages is needed to detect the target (Desmedt, 1980; Verleger, 1988).

The complexity analysis shows that FLOPs and Parameters using full-segment data are significantly higher than using single-segment data, which is due to the increase of input data volume and the increase of spatial-temporal graph convolution layers. However, the increased complexity remains in the same order of magnitude, so this is within an acceptable range.

### Study of Node Connection

**FIGURE 7:**
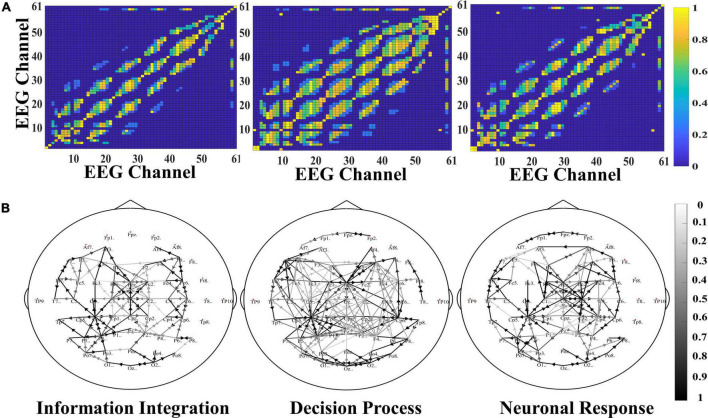
**(A)** Is the heatmap of adaptive adjacency matrix. **(B)** Is the brain network connection of three stages.

Here, the parameters of the SAST-GCN model are saved, and the learned adjacency matrix is calculated. To better display, the connection weight between the channels (matrix *M*) is transformed into the interval of [0,1], and a hot map is drawn in Figure 7A. In the figure, the closer the yellow blocks, the closer the connection between the electrodes; The closer the blue blocks, the sparser the connection between the electrodes. The results in the figure indicate that the adaptive spatial-temporal graph convolution can adaptively adjust the edge weights, make the network more flexible, and improve the performance of the model. Meanwhile, it is also proved that the connection between electrodes is not of the same importance, and different neighbor nodes can have different effects on the central node.

Figure 7B shows the brain network connection of three stages. The solid line in the figure represents the connection between the two electrodes, and the connection weight is greater than 0.8. The color of the line represents the connection strength between electrodes. The deeper the color of the line, the stronger the connection. The arrow in the figure represents the information flow direction. In the figure, it can be observed that the brain network connection in the decision process stage is the strongest, the number and density of connections is the largest, and the interaction is frequent in parietal lobe, occipital lobe, and frontal lobe regions. In addition, there is a synergistic interaction between multiple brain regions, and there are more long-range connections between multiple brain regions. However, in the information integration and neural response stages, the brain network connection is sparse and weak, and the connections are mainly small world connection. The information is mainly interactive between adjacent electrodes, mainly concentrated in the parietal lobe. From the changes of brain network in all three stages, there is a trend that brain network connection gradually enriched and then gradually weakened, which is consistent with the previous literature.

In addition, in order to explore the influence of different initialization methods of adjacency matrix on network performance, five other initial methods of adjacency matrix construction are used to represent the brain connection network. The five initial methods as comparison include fully connected matrix, random matrix, phase locking value matrix, coherence value matrix, physical distance matrix. Table 5 shows the effects of different initial adjacency matrixes on the results, including average accuracy, F1-score, recall and precious, which illustrates that the initial method using Pearson coefficient adjacency matrix is significantly better than other methods. Phase locking value matrix, coherence value matrix and physical distance matrix are better than random matrix and fully connected matrix, because the prior information is added. The performance of the fully connected matrix is the worst, which may be because it does not meet the sparsity requirement of the adjacency matrix.

**TABLE 5:** Comparison of effects of using different adjacency matrix initialization methods.

Method	Accuracy	F1-score	Precision	Recall
Fully connected matrix	0.8238	0.5251	0.4659	0.6202
Random matrix	0.8852	0.6589	0.6619	0.6726
Phase locking value matrix,	0.8861	0.6635	0.6507	0.6848
Coherence value matrix	0.8818	0.6512	0.6456	0.6677
Physical distance matrix	0.8880	0.6767	0.6779	0.6856
Pearson coefficient matrix	**0.9055**	**0.7042**	**0.7003**	**0.7100**

*The bold values represent the results of the proposed method.*

## Conclusion

Table 6 shows the SWOT analysis. In this paper, a graph neural network called SAST-GCN is proposed for single-trial video-induced P3 detection. The proposed model can extract the spatial-temporal characteristics of EEG data through adaptive spatial-temporal graph convolution according to different stages of video-induced P3. Also, it can learn the brain connection structure most suitable for ST-GCN to complete the detection task. In addition, the model introduces the style-based recalibration module to extract the spatial-temporal features with the highest contribution.

**TABLE 6:** SWOT analysis.

Strength	Weakness	Opportunity	Threat
1. An adjacency matrix is constructed to represent the brain network connection according to the neural mechanism of video processing	Disturbance of inter-subject difference on network performance	1. It is proved that the applicability of graph neural network for video-induced P3 detection	1. Uncertain perturbations of data need to be addressed
2. The spatial-temporal features of EEG data are extracted by adaptive spatial-temporal graph convolution		2. The construction of brain network connections by segments are better than those based on static graph design	2. The initialization method of adjacency matrix needs to be optimized.

In order to verify the performance of the proposed method, we compared the proposed method with the baseline, and the results showed that the proposed method was significantly superior to the baseline. Ablation experiments show that the segmented construction adjacency matrix operation and SRM have great contribution to improving the classification accuracy of the network. We also observed that the classification results using all stages were significantly better than using only one stage and using Pearson correlation coefficient to construct initial adjacency matrix than using other adjacency matrix initialization methods.

Our work proves that graph convolution based on the segmented constructing brain network connection is superior to the existing graph convolution based on static graph design. This is because the state of the brain network is dynamic rather than static. However, the dynamic variation of the brain network is not only reflected in the neural reaction stage, but also in the different subjects. In this paper, we average the ERP of all subjects, so as to determine the division of each stage, but there is a variation in latency of P3 between subjects, which may lead to different neural response stages of different subjects in time. Different segmentation strategies may be more appropriate for different subjects. The disturbance of data uncertainty between subjects will have a negative impact on the performance of the neural network. The fuzzy encoder can reduce the large fluctuation of the feature space to a narrower range of membership space, so the classifier based on fuzzy logic may reduce this adverse effect (Korytkowski et al., 2015; Versaci et al., 2020). On the other hand, this paper only discusses the method of constructing adjacency matrix by using the correlation between electrodes, however, for EEG data, probabilistic graph representation could be a better approach to describe the intrinsic relationships among the electrodes. In the future, our main work is to carry out the difference research between subjects and optimize the construction of brain network connections, so as to improve the robustness and fitting of the model.

## Data Availability Statement

The original contributions presented in the study are included in the article/supplementary material, further inquiries can be directed to the corresponding author/s.

## Author Contributions

RL was mainly responsible for research design, data analysis, and manuscript writing of this study. RZ was mainly responsible for data analysis and production of charts. YZ and BY were mainly responsible for research design. LT was mainly responsible for data collection and manuscript modification. All authors contributed to the article and approved the submitted version.

## Conflict of Interest

The authors declare that the research was conducted in the absence of any commercial or financial relationships that could be construed as a potential conflict of interest.

## Publisher’s Note

All claims expressed in this article are solely those of the authors and do not necessarily represent those of their affiliated organizations, or those of the publisher, the editors and the reviewers. Any product that may be evaluated in this article, or claim that may be made by its manufacturer, is not guaranteed or endorsed by the publisher.
